# Adesão ao Guia Alimentar da População Brasileira e aspectos sociodemográficos: Estudo Brazuca

**DOI:** 10.11606/s1518-8787.2025059006044

**Published:** 2025-05-26

**Authors:** Maria Eugênia de Medeiros Fernandes, Severina Carla Vieira Cunha Lima, Suamy Sales Barbosa, Rosa Sá de Oliveira Neta, Layanne Cristini Martin Sousa, Mariana Silva Bezerra, Márcia Marília Gomes Dantas Lopes, Angelo Giuseppe Roncalli da Costa Oliveira, Dirce Maria Lobo Marchioni, Clélia de Oliveira Lyra

**Affiliations:** I Universidade Federal do Rio Grande do Norte. Programa de Pós-graduação em Nutrição. Natal, RN, Brasil; II Universidade Federal do Rio Grande do Norte. Departamento de Nutrição. Natal, RN, Brasil; III Universidade Federal do Rio Grande do Norte. Programa de Pós-graduação em Saúde Coletiva. Natal, RN, Brasil; IV Universidade Federal da Bahia. Instituto Multidisciplinar em Saúde. Vitória da Conquista, BA, Brasil; V Universidade Federal do Rio Grande do Norte. Departamento de Odontologia. Natal, RN, Brasil; VI Universidade de São Paulo. Faculdade de Saúde Pública. Departamento de Nutrição. São Paulo, SP, Brasil

**Keywords:** Guias Alimentares, Práticas Alimentares Saudáveis, Dieta Saudável, Inquéritos Alimentares, Consumo Alimentar

## Abstract

Analisar a adesão às recomendações do Guia Alimentar e sua relação com fatores sociodemográficos entre adultos e idosos participantes do estudo Brazuca Natal.

Trata-se de uma pesquisa transversal, com 411 adultos e idosos do município de Natal, Rio Grande do Norte (RN), selecionados a partir de uma amostra probabilística por conglomerados, em dois estágios (setores censitários e domicílios). A coleta de dados foi realizada por questionário eletrônico na plataforma digital *Epicollect 5*, contendo dados sociodemográficos e uma escala multidimensional para mensuração da adesão às práticas alimentares recomendadas pelo Guia Alimentar para População Brasileira. A escala é composta por 24 perguntas, obedecendo uma escala *Likert* (discordo fortemente; discordo; concordo; concordo fortemente). O escore final pode variar de 0 a 72 e ser classificado como baixa adesão (< 32 pontos), média adesão (32 a 41 pontos), ou alta adesão ao Guia (> 41 pontos). Para verificar a associação entre a adesão ao Guia com as variáveis sociodemográficas, foi realizada análise múltipla por regressão logística incondicional.

O escore médio foi de 40,5 (7,9), e a alta adesão ao Guia foi observada em 40,8% (IC95% 30,8–51,5) da população, com associação significativa para o sexo feminino (RP = 1,27; IC95% 1,03–1,55) e pessoas idosas (RP = 1,46; IC95% 1,19–1,79). Pessoas que declararam renda *per capita* mensal inferior a um salário-mínimo obtiveram menor probabilidade de ter alta adesão ao Guia, fator intensificado para aqueles que ganhavam um valor ≤ 1/4 salário-mínimo (RP = 0,47; IC95% 0,32–0,68).

Ser do sexo feminino e ser idoso são condições que predizem maior adesão ao Guia, enquanto possuir uma baixa renda *per capita* prediz menor adesão na população avaliada. É necessário estabelecer políticas de redução às desigualdades sociais e ações para o maior acesso às práticas alimentares alinhadas ao Guia entre adultos e idosos.

## INTRODUÇÃO

As diretrizes ou guias alimentares de um país são documentos oficiais elaborados para nortear políticas públicas e orientar as pessoas sobre alimentação e saúde. A segunda edição do Guia Alimentar para População Brasileira (Guia), fornece um paradigma ampliado de alimentação saudável, uma vez que considera o tipo de processamento dos alimentos, a combinação e o preparo desses alimentos, as características do modo de comer e as dimensões socioculturais das práticas alimentares^1-3.^

O Guia também leva em conta os impactos na sociedade e meio ambiente gerados pelos sistemas alimentares e enfatiza a ampliação da autonomia das pessoas para escolhas alimentares saudáveis, superando limitações comuns das diretrizes alimentares convencionais^1,3,4^. As recomendações do Guia foram norteadas por princípios que fundamentam a proposição de recomendações, que consideram o cenário da epidemiologia nutricional no Brasil e sua relação com a alimentação adequada e saudável e a sustentabilidade do sistema alimentar; as recomendações gerais sobre a escolha de alimentos, o ato de comer e a comensalidade; e os fatores que podem ser obstáculos para a adesão das pessoas às recomendações do Guia. É essa multidimensionalidade que amplia o conceito de alimentação saudável adotado pelo Guia^3^.

As recomendações do Guia são baseadas também em uma classificação de alimentos que considera o processamento a que são submetidos (classificação Nova), sugerindo que seja priorizado o consumo de alimentos *in natura* ou minimamente processados e preparações culinárias com alimentos básicos e tradicionais em detrimento do consumo de alimentos ultraprocessados (AUP)^2,3^. Alimentos ultraprocessados, em geral, possuem composição nutricional inadequada e baixo valor nutricional quando comparados com alimentos minimamente processados (possuem maior densidade energética, mais açúcares livres, mais gorduras, em geral, e menos fibras), seu consumo é associado a desfechos negativos a saúde^5,6^.

O Brasil possui particularidades regionais, históricas e culturais que permitem um padrão alimentar, de pessoas adultas e idosas, ainda caracterizado pelo consumo de alimentos *in natura* ou minimamente processados, principalmente feijão e arroz, seguido de carnes (e leite pelas pessoas idosas), segundo dados da Pesquisa de Orçamentos Familiares (POF) de 2017–2018. No entanto, o consumo de AUP já contribui com quase 1/5 das calorias consumidas pelas pessoas adultas brasileiras e com cerca de 15% das calorias consumidas por pessoas idosas^7-9.^

Dado que o Guia orienta práticas alimentares saudáveis considerando suas múltiplas dimensões, avaliar a adesão de pessoas adultas e idosas as suas recomendações é um importante passo rumo à análise do impacto e complexidade desse instrumento. Segundo a Organização das Nações Unidas para Alimentação e Agricultura (FAO), a avaliação de guias alimentares é insuficiente em muitos países. Mensurar essa adesão torna possível dimensionar as características das práticas alimentares da população e as relações delas com outros determinantes (aspectos sociais, econômicos e demográficos, por exemplo)^2,10^.

Nesse sentido, optou-se por utilizar o modelo conceitual dos determinantes sociais da saúde da OMS para contextualizar alguns dos aspectos sociodemográficos (sexo, fase da vida, raça/cor da pele, situação conjugal, escolaridade e renda *per capita*) que podem estar relacionados à adesão de pessoas adultas e idosas às práticas alimentares recomendadas pelo Guia. Este modelo considera os determinantes estruturais (mecanismos sociais, econômicos e políticos) que atuam por meio de determinantes intermediários (circunstâncias materiais, fatores comportamentais, biológicos e psicossociais) para moldar efeitos na saúde^11^.

Considerando o desafio de avaliar a adesão das pessoas às recomendações do Guia, Gabe e Jaime^2^ desenvolveram e validaram uma escala multidimensional que permite tal objetivo. A escala é composta por 24 itens que exemplificam práticas alimentares alinhadas ou opostas às recomendações do Guia. Na literatura, poucos estudos utilizaram essa escala de adesão^12-14^. Posto a escassez de pesquisas que avaliem a adesão ao Guia entre pessoas adultas e idosas, o objetivo deste trabalho foi analisar a adesão às recomendações do Guia Alimentar e sua relação com fatores sociodemográficos entre pessoas adultas e idosas participantes do estudo *Brazilian Usual Consumption Assessment* (BRAZUCA) Natal. A execução deste estudo contribuirá para o monitoramento das práticas alimentares e diagnóstico da situação alimentar da população adulta e idosa em uma capital do nordeste do Brasil.

## MÉTODOS

Trata-se de um estudo transversal de base populacional. Este estudo é um recorte da pesquisa “Insegurança alimentar, condições de saúde e de nutrição em população adulta e idosa de uma capital do Nordeste do Brasil: Estudo Brazuca Natal”, de base populacional, com pessoas adultas e idosas do município de Natal/RN. Este estudo foi aprovado pelo Comitê de Ética em Pesquisa da Universidade Federal do Rio Grande do Norte (UFRN) (CAAE: 96294718.4.2001.5292), conforme as diretrizes regulamentadas da pesquisa envolvendo seres humanos (Resolução 466/12 do Conselho Nacional de Saúde).

O plano amostral do estudo Brazuca Natal foi realizado considerando uma amostra probabilística por conglomerados em dois estágios (setores censitários e domicílios). Os setores censitários foram sorteados com probabilidade proporcional ao tamanho (número de domicílios) e, antes do sorteio, foram ordenados segundo indicadores de escolaridade, derivados de informações do censo demográfico de 2010.

O sorteio foi realizado de forma a obter um mínimo de 258 entrevistas para cada um dos quatro estratos de sexo e idade: pessoas adultas (20 a 59 anos) e idosas (60 anos ou mais), ambos os sexos (feminino e masculino). O tamanho mínimo de 258 pessoas em cada estrato possibilita estimar uma prevalência de 50% para múltiplos desfechos relacionados a doenças ou agravos relacionados à nutrição, com erro de 8% e nível de confiança de 95%. O efeito de delineamento (*deff*) foi de 1,5 e foram acrescidos 15% como taxa de não-resposta e domicílios fechados. O tamanho total da amostra estimada foi de 1.032 pessoas.

Para este estudo, mediante a emergência de saúde pública do covid-19 e a consequente suspensão das coletas de dados do estudo Brazuca Natal, a população de estudo correspondeu a 38% dos setores censitários previstos para a coleta de dados. Foram incluídos 411 entrevistados no período de junho de 2019 a março de 2020. Para identificar um possível viés amostral, foi verificada a equivalência entre a amostra coletada e a estimada, procedendo-se os testes de análise das perdas dos setores censitários, comparando variáveis socioeconômicas e demográficas dos setores pesquisados e os não pesquisados. Foram testadas as variáveis “número de domicílios particulares permanentes”, “número de residentes dos domicílios particulares permanentes”, “média de moradores”, “rendimento nominal médio” e “razão de sexo”, por teste t e análise de valores omissos, considerando p < 0,05. A análise demonstrou que as perdas foram aleatórias (p = 0,135, teste MCAR de Little). O poder da amostra foi recalculado, considerando os seguintes parâmetros: população finita, como desfecho, a proporção de 40,8% das pessoas com alta adesão ao Guia; assumindo-se uma margem de erro absoluta de 4% e efeito do desenho de 1,5, o que resultou em um poder de 80%, com tamanho mínimo amostral de 372 pessoas.

A coleta de dados foi realizada nos domicílios, utilizando questionário padronizado em uma plataforma digital denominada Epicollect 5, e revisado a partir dos protocolos desenvolvidos especialmente para o estudo Brazuca Natal^15-17^, contendo dados socioeconômicos e demográficos, sendo eles: sexo, fase da vida, raça/cor da pele, situação conjugal, escolaridade e renda *per capita* (considerando que 1 salário-mínimo correspondia, em 2019, à R$ 998,00 ou US$ 240,98).

Para mensurar a adesão às práticas alimentares baseadas nas recomendações do Guia, foi utilizada uma escala multidimensional desenvolvida e validada por Gabe e Jaime^18^. A escala respondida pelos usuários compreende quatro dimensões do Guia – planejamento, organização doméstica, escolha dos alimentos, e modos de comer – representadas por um conjunto de 24 itens que exemplificam práticas alimentares alinhadas ou opostas às recomendações do Guia Alimentar. A cada item os respondentes deveriam indicar se endossavam ou não a prática no seu dia a dia, por meio de uma escala *Likert* de concordância com 4 pontos: discordo fortemente; discordo; concordo; concordo fortemente. O escore na escala é computado pela soma simples das respostas a esses itens (às quais são atribuídos valores de 0 a 3), que pode variar de 0 a 72 como valor máximo. Os 13 itens alinhados às recomendações do Guia são pontuados de modo que a resposta de máxima concordância seja a de maior valor (concordo fortemente = 3 pontos); já os 11 itens opostos às recomendações são pontuados de maneira inversa (discordo fortemente = 3 pontos). Para fins de comparação, optamos por utilizar os pontos de corte-proposto pelos autores: < P25 (< 32 pontos), classificado como “baixa adesão às práticas alimentares segundo as recomendações do Guia”; P25 até P75 (32 a 41 pontos), classificado como “média adesão às práticas alimentares segundo as recomendações do Guia”; e > P75 (> 41 pontos), classificado como “alta adesão às práticas alimentares segundo as recomendações do Guia”^2,18^.

Para análise dos dados foi construído um banco de dados no software *Statistical Package for the Social Sciences* (SPSS) versão 20, no qual foram categorizadas as variáveis. Análise descritiva das variáveis categóricas foram demonstradas por meio de frequências absolutas, percentuais e respectivos intervalos de confiança de 95% e o efeito do desenho para amostras complexas. No módulo de amostra complexa os valores percentuais e de associação foram ponderados por sexo, fase da vida e situação socioeconômica do setor censitário. Valores de efeito do desenho de até 2,5 foram considerados como estimativas precisas. A variável adesão às recomendações ao Guia foi corrigida para dados perdidos de quatro pessoas, com imputação pelo método *Preditive Mean Matching* (PMM), considerando o sexo (feminino, masculino); fase da vida (adulta ou idosa) e a escolaridade (não alfabetizado, ensino fundamental, ensino médio, ensino superior). A imputação de dados foi realizada com o intuito de minimizar ao máximo as perdas amostrais, principalmente na análise múltipla^19^.

**Figura 1.**
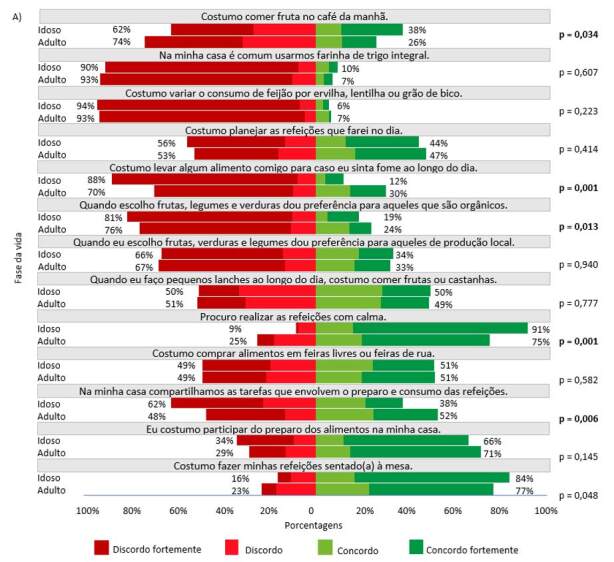
Distribuição das respostas aos itens da escala considerados práticas alimentares alinhadas as recomendações do Guia, segundo a fase da vida, Estudo Brazuca Natal – 2019–2020 (n = 411)

**Figura 2.**
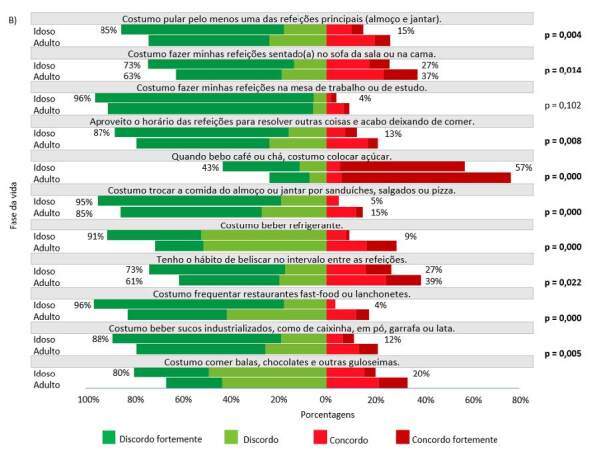
Distribuição das respostas aos itens da escala considerados práticas alimentares opostas as recomendações do Guia, segundo a fase da vida, Estudo Brazuca Natal – 2019–2020 (n = 411)

As práticas alimentares resultantes da escala de adesão ao Guia foram demonstradas em gráficos de barra, em duas figuras: Figura 1 com os 13 itens da escala alinhados às recomendações do Guia; e Figura 2 com os 11 itens opostos às recomendações do Guia. Para ambas, realizou-se o teste do qui-quadrado de Pearson para identificar associações entre as práticas alimentares e a fase da vida (adulta/idosa), uma vez que podem se alterar ao longo da vida. Para todas as associações com estratificação por idade verificou-se que o efeito do desenho foi inferior a 2,5 (dados não apresentados em tabelas ou figuras).

Para verificar a associação entre a adesão as práticas alimentares recomendadas pelo Guia com as variáveis demográficas e socioeconômicas, foi feita análise bivariada, considerando-se a adesão as práticas alimentares recomendadas pelo Guia (alta adesão e média/baixa adesão) como a variável dependente. As variáveis independentes foram: sexo (masculino e feminino), fase da vida (adulta e idosa), raça/cor da pele (branca e parda/negra/indígena), situação conjugal (com e sem companheiro), escolaridade (não alfabetizado, ensino fundamental, médio e superior), renda *per capita* (< ¼ de salário-mínimo (SM); ≥ ¼, < ½ SM; ≥ ½, < 1 SM e ≥ 1 SM) e acesso à água (diário e não diário). Utilizou-se o teste qui-quadrado de Pearson e foi demonstrado o efeito do desenho para amostras complexas. Utilizou-se o teste de qui-quadrado de tendência linear para avaliar a tendência da adesão ao Guia em relação ao aumento da renda *per capita*. Procedemos análise de modelagem múltipla com a finalidade de confirmar a associação entre prevalência de alta adesão às recomendações do Guia e todas as variáveis independentes. A seleção das variáveis explicativas para a construção do modelo múltiplo foi feita segundo critérios de plausibilidade biológica e estatísticos, dentre aquelas com valores de p < 0,20 para o teste do qui-quadrado de Pearson. Para esta análise foi utilizada a Regressão Logística incondicional e demonstrado os *odds ratio* (OR) brutos e ajustados. Optamos por incluir no modelo final as variáveis cujos valores de OR apresentaram intervalo de confiança significativos.

## RESULTADOS

Participaram do estudo 411 pessoas, com média de idade de 54,5 (16,9) anos. A maioria da população era do sexo feminino, adulta, que se declaram como parda/preta, residindo com companheiro(a) e apresentando ensino fundamental completo. Um percentual considerável dos entrevistados declarou renda per capita mensal de até ¼ do salário-mínimo. A proporção de alta adesão às recomendações do Guia foi de cerca de 40%, evidenciando situação preocupante (Tabela 1). O escore final da escala de práticas alimentares variou de 17 a 62 pontos, com média de 16,4 (5,5) referente as práticas alimentares alinhadas as recomendações do Guia e 23,8 (4,9) pontos correspondentes as práticas alimentares opostas as suas recomendações (dados não apresentados em tabelas).

**Tabela 1.** Características sociodemográficas e de adesão ao Guia, de pessoas adultas e idosas participantes do Estudo Brazuca Natal – 2019–2020 (n = 411).

Variáveis	% ^a^	IC95% ^a^	Efeito do desenho
Sexo			
Masculino	34,5	29,3–40,1	1,284
Feminino	65,5	59,9–70,7	
Fase da vida			
Adulta	88	85,1–90,3	0,616
Idosa	12	9,7–14,9	
Cor da pele			
Parda/Preta	65,5	55,8–74,0	3,628
Branca/Amarela	34,5	26,0–44,2	
Situação conjugal			
Com companheiro	62,7	55,2–69,7	2,213
Sem companheiro	37,3	30,3–44,8	
Escolaridade			
Não alfabetizado	3,7	1,9–7,2	1,731
Ensino fundamental	37,3	27,7–47,9	4,396
Ensino médio	37,7	31,7–44,0	1,606
Ensino superior	21,3	12,6–33,7	6,545
Renda *per capita*			
< ¼ salário–mínimo	21,5	13,4–32,7	5,457
≥ ¼ e < ½ salário–mínimo	22,7	15,8–31,5	3,484
≥ ½ e < 1 salário–mínimo	24,8	17,7–33,6	3,308
≥ 1 salário–mínimo	31,0	18,7–46,6	9,295
Acesso à água			
Não diário	20,5	14,8–27,7	2,507
Diário	79,5	72,3–85,2	
Adesão às recomendações do Guia			
Alta	40,8	30,8–51,5	4,459
Média	41,9	32,6–51,8	3,808
Baixa	17,4	12,2–24,1	2,390

IC95%: intervalo de confiança de 95%.

^a^Valores expressos em análise por amostragem complexa, ponderados por sexo, fase da vida e situação socioeconômica considerando o setor censitário.

**Tabela 2.** Análise bivariada da prevalência de alta adesão às recomendações do Guia com características demográficas e socioeconômicas de pessoas adultas e idosas participantes do Estudo Brazuca Natal – 2019–2020 (n = 411).

Variáveis/Categorias	Alta adesão	Média/baixa adesão	Efeito do desenho	p-valor
	n% (IC95%) ^a^	n% (IC95%) ^a^		
Sexo				0,076
Feminino	43,7 (32,8–55,3)	56,3 (44,7–67,2)	3,41	
Masculino	35,1 (24,5–47,5)	64,9 (52,5–75,5)	2,01	
Fase da vida				0,001
Idosa	59,6 (50,5–68,1)	40,4 (31,9–49,5)	0,39	
Adulta	38,2 (27,9–49,7)	61,8 (50,3–72,1)	4,49	
Cor da pele				0,293
Parda/Preta	38,4 (28,2–49,8)	61,6 (50,2–71,8	3,26	
Branca/Amarela	45,2 (31,8–59,3)	54,8 (40,7–68,2)	2,71	
Situação conjugal				0,182
Sem companheiro	35,2 (25,8–45,9)	64,8 (54,1–74,2)	1,65	
Com companheiro	44,1 (31,2–57,7)	55,9 (42,3–68,8)	4,59	
Escolaridade ^b^				0,005
Não alfabetizado	30,5 (18,4–46,1)	69,5 (53,9–81,6)	0,34	
Ensino fundamental	33,0 (22,2–46,1)	67,0 (53,9–77,8)	2,44	
Ensino médio	36,6 (24,1–51,2)	63,4 (48,8–75,9)	3,05	
Ensino superior	63,4 (47,1–77,2)	36,6 (22,8–52,9)	2,16	
Renda *per capita* ^c^				0,009
< ¼ salário-mínimo	25,1 (17,7–34,4)	74,9 (65,6–82,3)	0,78	
≥ ¼ e < ½ salário-mínimo	37,3 (22,6–54,9)	62,7 (45,1–77,4)	2,65	
≥ ½ e < 1 salário-mínimo	39,5 (26,2–54,5)	60,5 (45,5–73,8)	2,15	
≥ 1 salário-mínimo	55,2 (42,3–67,4)	44,8 (32,6–57,7)	2,03	
Acesso à água				0,065
Não diário	29,1 (16,8–45,3)	70,9 (54,7–83,2)	2,06	
Diário	43,8 (33,4–54,8)	56,2 (45,2–66,6)	3,73	

IC95%: intervalo de confiança de 95%.

^a^Valores expressos em análise por amostragem complexa, ponderados por sexo, fase da vida e situação socioeconômica considerando o setor censitário.

^b^Teste qui-quadrado de tendência linear = 4,36 (p = 0,0037).

^c^Teste qui-quadrado de tendência linear = 4,72 (p = 0,029).

Nas Figuras 1 e 2 estão descritos, separadamente, cada item da escala com as respectivas respostas dos entrevistados (pessoas adultas e idosas), para demonstrar as diferenças entre essas fases da vida. A cor verde representa as práticas alimentares desejáveis (alinhadas às recomendações do Guia) e a cor vermelha o oposto. A Figura 1 mostra a distribuição das respostas, aos itens que correspondem as práticas alimentares alinhadas as recomendações do Guia (dimensões “organização doméstica” e “planejamento”), por fase da vida (adulta e idosa). O recomendado é que os entrevistados concordem com as afirmações (cores verdes). De acordo com o gráfico, foram verificadas associações significativas entre a população adulta e idosa (p ≤ 0,05), nas questões referentes à “Costumo comer frutas no café da manhã”, “Costumo levar algo comigo caso sinta fome ao longo do dia”, “Quando escolho frutas, legumes e verduras, dou preferência aos que são orgânicos”, “Procuro realizar as refeições com calma” e “Na minha casa, compartilhamos tarefas que envolvem o preparo e consumo de refeições”.

Nota: p = valor de p referente ao teste de qui-quadrado de Pearson.

Nota: p = valor de p referente ao teste de qui-quadrado de Pearson.

A Figura 2 apresenta a distribuição das respostas aos itens referentes às práticas alimentares opostas as recomendações do Guia (dimensões “escolha dos alimentos” e “modos de comer”), por fase da vida (adulta e idosa), em que o recomendado é que os entrevistados discordem das afirmações (cor verde). Foram encontradas diferenças significativas entre as fases da vida (p ≤ 0,05), mostrando que as pessoas idosas apresentam maior proporção de práticas alinhadas ao Guia em todos os itens, com exceção à “Costumo fazer minhas refeições na mesa de trabalho ou estudo”.

Considerando o desfecho da prevalência de adesão as práticas alimentares alinhadas as recomendações do Guia, a Tabela 2 apresenta o resultado da análise bivariada com fatores demográficos e socioeconômicos. Considerando-se a alta adesão como a categoria de análise, o sexo feminino apresentou tendência a maior adesão as recomendações do Guia (p = 0,076), e foi associada negativamente para aqueles que não tinham acesso diário à água (p = 0,0065). A alta adesão foi maior na população idosa (p = 0,001), e quanto maior a escolaridade e a renda *per capita*, maior a adesão (conforme qui-quadrado de tendência linear 4,36 (p = 0,0037); e 4,72 (p = 0,029) respectivamente).

**Tabela 3.** Análise múltipla da prevalência de alta adesão às recomendações do Guia com características demográficas e socioeconômicas de pessoas adultas e idosas participantes do Estudo Brazuca Natal – 2019–2020 (n = 411).

Variáveis/categorias	OR bruto ^a^	OR ajustado ^a^
	(IC95%)	(IC95%)
Sexo		
Feminino	1,44 (0,96–2,15)	1,70 (1,10–2,64)
Masculino	1,00	1,00
Fase da vida		
Idosa	2,39 (1,47–3,88)	2,27 (1,36–3,80)
Adulta	1,00	1,00
Renda *per capita* ^b^		
< ¼ salário-mínimo	0,43 (0,25–0,73)	0,25 (0,12–0,53)
≥ ¼ e < ½ salário-mínimo	0,94 (0,67–1,33)	0,45 (0,18–1,14)
≥ ½ e < 1 salário-mínimo	1,65 (1,12–2,43)	0,51 (0,27–0,97)
≥ 1 salário-mínimo	1,00	1,00

IC95%; intervalo de confiança de 95%.

^a^Valores expressos em análise por amostragem complexa, ponderados por sexo, fase da vida e situação socioeconômica considerando o setor censitário.

^b^Teste qui-quadrado de tendência linear = 4,72 (p = 0,029).

A Tabela 3 apresenta o resultado da análise múltipla com o modelo final significativo que confirmou que a alta adesão foi maior entre pessoas do sexo feminino, entre pessoas idosas e entre aquelas com renda *per capita* superior a um salário-mínimo.

## DISCUSSÃO

Nosso estudo analisou a adesão de uma população amostral de pessoas adultas e idosas residentes em uma capital do Nordeste brasileiro, às recomendações de práticas alimentares do Guia^2^, sendo um dos primeiros estudos com tal intuito realizado nessa região. Optamos por avaliar a condição de alta adesão as práticas alimentares recomendadas pelo Guia como desfecho, para entender e incentivar as práticas que a caracterizam e para conhecer quem são as pessoas que podem estar mais vulneráveis (baixa adesão). Nesse sentido, a alta adesão associou-se positivamente ao sexo feminino e às pessoas idosas, enquanto àquelas com renda *per capita* de até ¼ do salário-mínimo apresentaram menor probabilidade de alta adesão. Relativo ao escore geral, menos da metade da população apresentou alta adesão.

A distribuição das respostas por fase da vida aos itens da escala endossa algumas reflexões. O achado em que as pessoas adultas discordaram mais que as idosas em relação à sentença “Costumo comer frutas no café da manhã”, demonstra uma convergência com o achado da POF 2017–2018: o consumo de frutas, verduras e legumes nessa fase da vida, apresentou uma queda quando comparado a anos anteriores, e continua muito aquém do recomendado^8,9^.

Chama-se atenção à grande discordância das pessoas, tanto adultas quanto idosas, para as sentenças “Na minha casa é comum usarmos farinha de trigo integral” e “Costumo variar o consumo de feijão por ervilha, lentilha ou grão de bico”. Esse resultado pode ser fruto de uma particularidade regional: no Nordeste, o hábito de consumo desses alimentos não é comum. No entanto, uma variedade de alimentos *in natura* e minimamente processados encontrados nessa região pode refletir um consumo adequado de fibras – utilizando-se alimentos como por exemplo, farinha de mandioca, cuscuz e raízes regionais –, bem como de leguminosas, considerando os diferentes tipos de feijões. A região Nordeste possui uma culinária característica, com alimentos regionais considerados boas fontes de diversos nutrientes, destacando-se a variedade de leguminosas (algaroba, feijão, feijão-de-corda, feijão-verde, guandu), tubérculos, raízes e cereais (araruta, gergelim, inhame, junça, mandioca, sorgo) e farinhas (farinha de tapioca)^20^. A escala de adesão ao Guia considera a diversidade de leguminosas, exemplificando com variedades distintas como grão de bico, ervilhas e lentilhas, que podem não refletir necessariamente o hábito local e, portanto, as respostas a essa questão podem não refletir a realidade da população nordestina. Nesse sentido, adequações nas orientações para aplicação da escala considerando as particularidades alimentares das diferentes regiões do país poderiam engrandecer essa importante ferramenta.

Um fator positivo pode ser observado no alto percentual de respostas concordantes, especialmente pelas pessoas idosas, aos itens “Procuro realizar as refeições com calma” e “Na minha casa compartilhamos as tarefas que envolvem o preparo e consumo das refeições”, sinalizando a importância dada aos modos de comer e compartilhamento das refeições nesta fase da vida. Ao contrário, pessoas adultas ao concordarem mais que as idosas com algumas práticas relacionadas a comportamentos e modos de comer inapropriados, como por exemplo “Costumo fazer minhas refeições sentado(a) no sofá da sala ou cama” “Aproveito o horário das refeições para resolver outras coisas e acabo deixando de comer” e “Tenho o hábito de beliscar no intervalo entre as refeições”, evidenciam alguns possíveis obstáculos para adoção de uma alimentação mais saudável no grupo de adultos, tais como: falta de tempo para organização e preparo dos alimentos, a falta de habilidade culinária ou a sobrecarga de tarefas ligadas à alimentação sobre uma única pessoa da família. É de conhecimento científico que modos de comer inapropriados como alimentar-se durante a realização de outras atividades, em qualquer espaço e o distanciamento da tradição culinária podem facilmente favorecer o consumo de AUP, que são projetadas para serem acessíveis, convenientes e hiper palatáveis^2,18,21^.

Em paralelo, um percentual considerável dessas pessoas adultas também concorda mais que as pessoas idosas com questões relacionadas ao consumo desses alimentos (AUP), tais como “Costumo beber refrigerante”, “Costumo beber sucos industrializados, como de caixinha, em pó, garrafa ou lata” e “Costumo comer balas, chocolates e outras guloseimas. Segundo dados da POF 2017–2018^8^, os AUP já contribuem com 19,5% das calorias consumidas pelas pessoas adultas brasileiras, com destaque para biscoito salgado e salgadinho”de pacote”, pães industrializados, biscoitos doces e frios e embutidos. Um destaque pode ser dado ao elevado percentual de pessoas idosas que também concordou com as afirmações “Quando bebo café ou chá, costumo colocar açúcar” e “Costumo comer balas, chocolates e outras guloseimas”. Os AUP já contribuem com cerca de 15% das calorias consumidas por essas pessoas, com destaque para bolachas salgadas e pães industrializados, seguidos dos doces e guloseimas^8^.

Os resultados de alta adesão em pessoas idosas e menor dentre aquelas com renda *per capita* de até ¼ do salário mínimo são semelhantes aos de Gabe e Jaime^2^, que também encontraram associação direta entre a adequação às recomendações do Guia e ao aumento da idade, e tendência de redução da adesão no sentido das classes econômicas mais altas para as mais baixas. A linearidade entre o aumento da idade e melhores práticas alimentares pode ser reflexo de fatores socioeconômicos (rendas fixas e permanentes provenientes de aposentadorias, pensões e benefícios sociais), estilo de vida e maior disponibilidade para o preparo das refeições, ou ainda, da presença de doenças crônicas e comorbidades que exigem a adoção de hábitos alimentares mais saudáveis. Vale lembrar que a saúde das pessoas idosas é multidimensional e que a melhora em uma dimensão (a alimentação, por exemplo) não se reflete necessariamente em outra^22^.

Com relação a maior probabilidade, neste estudo, de alta adesão as recomendações do Guia no sexo feminino, temos que o estilo de vida e as atitudes relacionadas à escolha de alimentos pelas mulheres podem influenciar as práticas alimentares. Um estudo observou que o comportamento relacionado a um padrão alimentar saudável, denominado protetor, foi maior entre mulheres brancas e com mais idade^23^. As habilidades culinárias entre as mulheres também podem influenciar neste resultado. De modo complementar, outro estudo verificou maior consumo de frutas por mulheres, apresentando uma relação inversamente proporcional com a ingestão de AUP^24^.

O achado que relaciona pessoas com menor renda *per capita* a uma prevalência menor de alta adesão ao Guia evidencia a importância de políticas voltadas para a melhoria das condições de vida dessas pessoas. No Brasil, embora o consumo de AUP seja menor nos quintos mais baixos de renda familiar, a presença desses alimentos permeia todas as camadas sociais^9^. A dificuldade de adquirir itens básicos considerados saudáveis como frutas, verduras, alimentos integrais e oleaginosas podem interferir diretamente na adoção de uma alimentação saudável^25^. No mais, o consumo alimentar perpassa a qualidade do alimento, e é influenciado por características culturais, históricas e psicológicas, que estão atreladas a situação de baixa renda^9^.

Foram consideradas limitações inerentes ao instrumento o fato de pessoas idosas não terem sido incluídas no estudo de desenvolvimento e validação da escala de adesão as recomendações do Guia. Outra limitação se dá em função da população ser homogênea quanto à renda *per capita* – grande parte das pessoas incluídas no estudo possuírem baixa renda. Ademais, os valores de efeito do desenho para algumas estimativas podem indicar menor precisão das estimativas e são diferentes para cada desfecho. Isso pode ter ocorrido por questões da suspensão da coleta de dados devido a covid-19. No entanto, esses fatos não invalidam os achados, uma vez que mostramos as peculiaridades do instrumento para pessoas idosas e em relação aos hábitos culturais de uma capital do Nordeste do Brasil, e fizemos um estudo criterioso quanto à análise de perdas. Outro ponto a ser considerado é que optamos em utilizar os pontos de corte dos autores para a escala de adesão ao Guia, e, portanto, foi considerado os percentis de outra amostra. No entanto ressaltamos que isso não invalida nossos achados, considerando que a escala foi validada para pessoas adultas e idosas brasileiras^26^.

Salientamos outra potencialidade do estudo, que foi evidenciar a violação do direito humano à alimentação adequada e saudável e outros direitos, da grande maioria da população que vive com baixa renda no município antes da pandemia. Destaca-se também que este é um dos primeiros estudos no nordeste do Brasil a utilizar a escala multidimensional para avaliação de práticas alimentares de acordo com o Guia.

O estudo demonstrou que existe melhor adesão ao Guia entre mulheres e pessoas idosas, que comumente tem maior preocupação e cuidado com a saúde no Brasil. Ademais, ficou evidente que a menor renda parece ser um determinante para pior adesão às recomendações do Guia. Esta pesquisa corrobora o potencial dessa ferramenta para orientar políticas públicas de promoção da alimentação saudável e podem ser úteis para direcionar ações em nível local e nacional, de disseminação do Guia.
