# Supplementary material for: Adesão ao Guia Alimentar da População Brasileira e aspectos sociodemográficos: Estudo Brazuca
Source: Rev Saude Publica. 2025 May 26;59:e16. [Article in Portuguese] doi: 10.11606/s1518-8787.2025059006044 (PMC12112441; doi:10.11606/s1518-8787.2025059006044)
Supplement: Translation

# Translation: Adherence to the Dietary Guideline for the Brazilian Population and its sociodemographic aspects: Brazuca Natal Study

**Authors:** Maria Eugênia de Medeiros Fernandes, Severina Carla Vieira Cunha Lima, Suamy Sales Barbosa, Rosa Sá de Oliveira Neta, Layanne Cristini Martin Sousa, Mariana Silva Bezerra, Márcia Marília Gomes Dantas Lopes, Angelo Giuseppe Roncalli da Costa Oliveira, Dirce Maria Lobo Marchioni, Clélia de Oliveira Lyra

**Affiliations:** I Universidade Federal do Rio Grande do Norte. Programa de Pós-graduação em Nutrição. Natal, RN, Brasil; II Universidade Federal do Rio Grande do Norte. Departamento de Nutrição. Natal, RN, Brasil; III Universidade Federal do Rio Grande do Norte. Programa de Pós-graduação em Saúde Coletiva. Natal, RN, Brasil; IV Universidade Federal da Bahia. Instituto Multidisciplinar em Saúde. Vitória da Conquista, BA, Brasil; V Universidade Federal do Rio Grande do Norte. Departamento de Odontologia. Natal, RN, Brasil; VI Universidade de São Paulo. Faculdade de Saúde Pública. Departamento de Nutrição. São Paulo, SP, Brasil

**Keywords:** Food Guides, Healthy Eating Practices, Healthy Diet, Food Surveys, Food Consumption

## Abstract

To analyze the adherence of adults and older adults in the Brazuca Natal study to the recommendations of the Dietary Guideline for the Brazilian Population and to determine its relation with sociodemographic factors.

This cross-sectional study was carried out with 411 adults and older individuals residing in Natal, Rio Grande do Norte. Participants were selected from a probabilistic sample by clusters in two stages (census tracts and households). Data were collected by an electronic questionnaire on the Epicollect 5 digital platform. The questionnaire contained sociodemographic data and a multidimensional scale to measure adherence to the dietary practices recommended by the Dietary Guidelines for the Brazilian population. The scale consists of 24 questions following a Likert scale (strongly disagree; disagree; agree; and strongly agree). Total scores ranged from 0 to 72, with classifications of low (total score < 32), medium (total score from 32 to 41), and high adherence (total score > 41) to the Dietary Guideline. To verify the association between adherence to the Guide and sociodemographic variables, multiple analysis by unconditional logistic regression were performed.

The mean score equaled 40.5 (7.9), and high adherence to the Guide occurred in 40.8% (95%CI 30.8–51.5) of the population. Women (PR = 1.27; 95%CI 1.03–1.55) and older individuals (PR = 1.46; 95%CI 1.19–1.79) showed a significant association. Individuals who reported a monthly *per capita* income below one minimum wage were less likely to show high adherence to the Guide, a factor that was intensified for those who earned ≤ 1/4 of the minimum wage (PR = 0.47; 95%CI 0.32–0.68).

The findings of this study indicate that women and older age predict greater adherence to the Dietary Guideline. Conversely, having a low per capita income predicts alower adherence in the evaluated population. Establishing policies to mitigate social inequalities and initiatives to enhance access to dietary practices in line with the Dietary Guideline among adult and older adult populations is imperative.

## INTRODUCTION

The food guidelines or guides of a country constitute official documents that aim to direct public policies and guide people on food and health. The second edition of the Dietary Guidelines for the Brazilian Population (Guide) provides an expanded paradigm of healthy eating since it considers the type of food processing, the combination and preparation of these foods, the characteristics of manners of eating, and the sociocultural dimensions of eating practices^1,3^

The Guide also considers the impacts of food systems on society and the environment and stresses the expansion of people’s autonomy toward healthy food choices, overcoming common limitations to conventional dietary guidelines^1,3,4^. Its recommendations follow principles that consider the scenario of nutritional epidemiology in Brazil, its relation with adequate and healthy eating, the sustainability of the food system; general recommendations on food choice, eating, and commensality; and the factors that may hinder people’s adherence to these recommendations. Such multidimensionality expands the concept of healthy eating in the Guide^3^.

The Guide recommendations stem from a food classification that considers the processing to which they are subjected (the Nova classification), suggesting the prioritization of fresh or minimally processed foods and culinary preparations with basic and traditional foods to the detriment of ultra-processed foods (UPF)^2,3^. Ultra-processed foods generally have poorer nutritional composition and lower nutritional value than minimally processed foods due to their higher energy density, more free sugars and fats, and generally less fiber. Moreover, their consumption is associated with negative health outcomes^5,6^.

The regional, historical, and cultural particularities of Brazil constitute a dietary pattern in adults and older adults that remains marked by the consumption of fresh or minimally processed foods, especially beans and rice, followed by meat (and milk by older adults), according to data from the 2017–2018 *Pesquisa de Orçamentos Familiares* (POF – Brazilian Consumer Expenditure Survey). However, the consumption of UPF contributes to almost 1/5 of the calories Brazilian adults consume and about 15% of that in older adults^7-9^.

As the Guide suggests healthy eating practices considering its multiple dimensions, assessing the adherence of adults and older adults to its recommendations constitutes an important step to analyze the impact and complexity of this instrument. According to the Food and Agriculture Organization of the United Nations, many countries fail to properly evaluate their food guides. Quantifying this adherence can measure the characteristics of populations’ eating practices and their relations with other determinants (social, economic, and demographic aspects, for example)^2,10^.

Thus, we chose to use the WHO conceptual model of social determinants of health to contextualize some of the sociodemographic aspects (sex, stage of life, race/skin color, marital status, education, and *per capita* income) that may be related to the adherence of adults and older adults to the dietary practices in the Guide. This model considers the structural determinants (social, economic, and political mechanisms) that act by intermediate determinants (material circumstances, behavioral, biological, and psychosocial factors) to shape health effects^11^.

Considering the challenge of assessing people’s adherence to the Guide recommendations, Gabe and Jaime^2^ developed and validated a multidimensional scale for such objective. The scale consists of 24 items that exemplify dietary practices in line with the recommendations of the Guide or opposed to it. Few studies in the literature have used this adherence scale^12-14^. Given the scarcity of studies that evaluate adherence to the Guide in adults and older adults, this study aimed to analyze adherence to the recommendations of the Guide and its relationship with sociodemographic factors in adult participants in the Brazilian Usual Consumption Assessment (BRAZUCA) Natal study. This study will contribute to monitoring feeding practices and the diagnosis of the dietary situation of adults and older adults in a northeastern Brazilian capital.

## METHODS

This is a population-based cross-sectional study that stems from the research “*Insegurança alimentar, condições de saúde e de nutrição em população adulta e idosa de uma capital do Nordeste do Brasil: Estudo Brazuca Natal*” with adults and older adults in the municipality of Natal, in the state of Rio Grande do Norte. This study was approved by the Research Ethics Committee of Universidade Federal do Rio Grande do Norte (CAAE: 96294718.4.2001.5292) in accordance with the regulated guidelines for research involving human beings (Resolution 466/12 of the National Health Council).

The Brazuca Natal study was carried out considering a two-stage (census tracts and households) probabilistic cluster sample. The census tracts were drawn with probability proportional to their size (number of households) and, before the draw, they were ordered according to schooling indicators based on information from the 2010 demographic census.

The draw was carried out to obtain a minimum of 258 interviews for each of four sex and age strata: adults (aged from 20 to 59 years) and older adults (aged from 60 years or above) of all sexes (female and male). The minimum size of 258 people in each stratum estimated a prevalence of 50% for multiple outcomes related to nutrition-related diseases or conditions, with an error of 8% and a confidence level of 95%. The design effect totaled 1.5, to which 15% were added as the rate of non-response and closed households. The total estimated sample size equaled 1,032 people.

Due to the public health emergency of COVID-19 and the consequent suspension of data collection from the Brazuca Natal study, the population of this study corresponded to 38% of the census tracts planned for data collection. A total of 411 respondents were included from June 2019 to March 2020. To find a possible sampling bias, the equivalence between the collected and estimated samples was assessed by tests to analyze census tract losses by comparing socioeconomic and demographic variables of the surveyed and non-surveyed tracts. The variables “number of permanent private households,” “number of residents of permanent private households,” “average number of residents,” “average nominal income,” and “sex ratio” were tested by t-test and missing value analysis at p < 0.05. Analysis showed random losses (p = 0.135, Little’s MCAR test). The power of the sample was recalculated considering the following parameters: finite population as an outcome, the proportion of 40.8% of people with high adherence to the Guide; assuming an absolute margin of error of 4% and design effect of 1.5, which resulted in a power of 80% and a minimum sample size of 372 people.

Data were collected from the households by a standardized questionnaire on a digital platform called Epicollect 5 and reviewed based on protocols that had been developed especially for the Brazuca Natal study^15-17^, containing socioeconomic and demographic data, namely: sex, stage of life, race/skin color, marital status, education, and *per capita* income (considering that one minimum wage corresponded to R$ 998.00 or US$ 240.98 in 2019).

A multidimensional scale developed and validated by Gabe and Jaime^18^ was used to measure adherence to dietary practices following Guide recommendations. The scale answered by users comprises four Guide dimensions—planning, household organization, choice of food, and manners of eating—represented by a set of 24 items that exemplify eating practices in line with Guide recommendations or opposed to it. For each item, respondents had to indicate whether they endorsed the practice in their daily lives by a four-point Likert scale: Strongly disagree; Disagree; Agree; and Strongly agree. The scale score is computed by summing the answers to these items (to which values from 0 to 3 are assigned), which can vary from 0 to 72. The 13 items in line with Guide recommendations are scored so that the answer with maximum agreement has the highest value (strongly agree = three points), whereas the 11 items opposed to the recommendations are scored inversely (strongly disagree = three points). The cut-off points the authors proposed were used for comparison: < P25 (< 32 points), classified as “low adherence to dietary practices according to Guide recommendations;” P25 to P75 (32 to 41 points), classified as “medium adherence to dietary practices according to Guide recommendations;” and > P75 (> 41 points); classified as “high adherence to dietary practices according to Guide recommendations”^2,18^.

For data analysis, a database was built on the Statistical Package for the Social Sciences, version 20, in which the variables were categorized. The descriptive analysis of categorical variables is shown as absolute frequencies, percentages, and their respective 95% confidence intervals and the effect of the design for complex samples. In the complex sample module, the percentage and association values were weighted by sex, stage of life, and socioeconomic status of the census tract. Design effect values of up to 2.5 were considered as accurate estimates. The variable adherence to Guide recommendations was corrected for lost data of four people, with imputation by the predictive mean matching method, considering sex (female, male); stage of life (adult or older adult), and education (illiterate, primary education, secondary education, higher education). Data was imputed to minimize sample losses as much as possible, especially in the multiple analysis^19^.

The dietary practices from the scale of adherence to the Guide are shown in bar graphs in two figures: Figure 1 shows the 13 items of the scale in line with Guide recommendations and Figure 2, the 11 items opposed to them. For both, the Pearson’s chi-squared test was performed to find associations between dietary practices and the stage of life (adult/older adult) since they can change throughout life. All associations with stratification by age show an effect of the design below 2.5 (data not shown in tables or figures).

To test the association between adherence to the dietary practices recommended by the Guide with the chosen demographic and socioeconomic variables, a bivariate analysis was performed considering adherence to the dietary practices recommended by the Guide (high and medium/low adherence) as the dependent variable. The following were chosen as independent variables: sex (male and female), life stage (adult and older adult), race/skin color (White and Brown/Black/Indigenous), marital status (with and without a partner), education (illiterate, primary, secondary, and higher education), per capita income (< ¼ of a minimum wage; ≥ ¼, < ½ of a minimum wage; ≥ ½, < 1 of a minimum wage, and ≥ 1 minimum wage), and access to water (daily and non-daily). Pearson’s chi-squared test was used and the design effect for complex samples was shown. The chi-squared test of linear trend was used to assess the trend of adherence to the Guide regarding the increase in *per capita* income. We performed multiple modeling analysis to confirm the association between prevalence of high adherence to Guide recommendations and all independent variables. The explanatory variables were chosen for the multiple model according to biological and statistical plausibility criteria from those with p-values < 0.20 for Pearson’s chi-squared test. An unconditional logistic regression and gross and adjusted odds ratio (OR) were used in this analysis. The variables the OR values of which showed significant confidence intervals were inputted into the final model.

## RESULTS

This study included 411 people, the mean age of whom equaled 54.5 (16.9) years. Most were women, adults, declared themselves as Brown/Black, lived with a partner, and had completed primary education. A considerable percentage of interviewees declared a monthly *per capita* income of up to a fourth of the Brazilian minimum wage. The proportion of high adherence to Guide recommendations equaled about 40%, evincing a worrying situation (Table 1). The final score of the eating practices scale ranged from 17 to 62 points, with a mean of 16.4 (5.5) points for eating practices in line with Guide recommendations and 23.8 (4.9) points for those opposed to them (data not shown in tables).

**Table 1.** Sociodemographic characteristics and adherence to the Guide in adults and older adults in the Brazuca Natal study – 2019–2020 (n = 411).

Variables	% ^a^	95%CI ^a^	Design effect
Sex			
Male	34.5	29.3–40.1	1.284
Female	65.5	59.9–70.7	
Stage of life			
Adult	88	85.1–90.3	0.616
Older adult	12	9.7–14.9	
Skin color			
Black/mixed-race	65.5	55.8–74.0	3.628
White/Yellow	34.5	26.0–44.2	
Marital status			
With a partner	62.7	55.2–69.7	2.213
Without a partner	37.3	30.3–44.8	
Schooling			
Illiterate	3.7	1.9–7.2	1.731
Primary education	37.3	27.7–47.9	4.396
Secondary education	37.7	31.7–44.0	1.606
Tertiary education	21.3	12.6–33.7	6.545
*Per capita* income			
< ¼ minimum wage	21.5	13.4–32.7	5.457
≥ ¼ and < ½ minimum wage	22.7	15.8–31.5	3.484
≥ ½ and < 1 minimum wage	24.8	17.7–33.6	3.308
≥ 1 minimum wage	31.0	18.7–46.6	9.295
Access to water			
Non-daily	20.5	14.8–27.7	2.507
Daily	79.5	72.3–85.2	
Adherence to Guide recommendations			
High	40.8	30.8–51.5	4.459
Medium	41.9	32.6–51.8	3.808
Low	17.4	12.2–24.1	2.390

95%CI: 95% confidence interval.

^a^Values expressed in complex sampling analysis, weighted by sex, life stage, and socioeconomic status considering the census tract.

**Table 2.** Bivariate analysis of the prevalence of high adherence to Guide recommendations with demographic and socioeconomic characteristics of adults and older adults in the Brazuca Natal study – 2019–2020 (n = 411).

Variables/Categories	High adherence	Medium/low adherence	Design effect	p-value
	n% (95%CI) ^a^	n% (95%CI) ^a^		
Sex				0.076
Female	43.7 (32.8–55.3)	56.3 (44.7–67.2)	3.41	
Male	35.1 (24.5–47.5)	64.9 (52.5–75.5)	2.01	
Stage of life				0.001
Older adult	59.6 (50.5–68.1)	40.4 (31.9–49.5)	0.39	
Adult	38.2 (27.9–49.7)	61.8 (50.3–72.1)	4.49	
Skin color				0.293
Black/mixed-race	38.4 (28.2–49.8)	61.6 (50.2–71.8	3.26	
White/Yellow	45.2 (31.8–59.3)	54.8 (40.7–68.2)	2.71	
Marital status				0.182
Without a partner	35.2 (25.8–45.9)	64.8 (54.1–74.2)	1.65	
With a partner	44.1 (31.2–57.7)	55.9 (42.3–68.8)	4.59	
Schooling ^b^				0.005
Illiterate	30.5 (18.4–46.1)	69.5 (53.9–81.6)	0.34	
Primary education	33.0 (22.2–46.1)	67.0 (53.9–77.8)	2.44	
Secondary education	36.6 (24.1–51.2)	63.4 (48.8–75.9)	3.05	
Tertiary education	63.4 (47.1–77.2)	36.6 (22.8–52.9)	2.16	
*Per capita* income ^c^				0.009
< ¼ minimum wage	25.1 (17.7–34.4)	74.9 (65.6–82.3)	0.78	
≥ ¼ and < ½ minimum wage	37.3 (22.6–54.9)	62.7 (45.1–77.4)	2.65	
≥ ½ and < 1 minimum wage	39.5 (26.2–54.5)	60.5 (45.5–73.8)	2.15	
≥ 1 minimum wage	55.2 (42.3–67.4)	44.8 (32.6–57.7)	2.03	
Access to water				0.065
Non-daily	29.1 (16.8–45.3)	70.9 (54.7–83.2)	2.06	
Daily	43.8 (33.4–54.8)	56.2 (45.2–66.6)	3.73	

95%CI: 95% confidence interval

^a^Values expressed in complex sampling analysis, weighted by sex, life stage, and socioeconomic status considering the census tract.

^b^Chi-squared test of linear trend = 4.36 (p = 0.0037).

^c^Chi-squared test of linear trend = 4.72 (p = 0.029).

Figures 1 and 2 describe each item of the scale and interviewees’ (adults and older adults) answers to them to show the differences between these stages of life. Green represents desirable eating practices (in line with Guide recommendations) and red, the opposite. Figure 1 shows the distribution of the answers to the items that correspond to the dietary practices in line with Guide recommendations (the “domestic organization” and “planning” dimensions) by stage of life (adult and older adult). It is recommended that the interviewees agree with the statements (green colors). The graph shows significant associations between the adult and older adult population (p ≤ 0.05) in the following questions: “I usually eat fruit for breakfast,” “I usually take something with me if I feel hungry throughout the day,” “When I choose fruits, vegetables, and greens, I prefer organic ones,” “I try to eat meals slowly,” and “At home, we share the tasks involved in preparing and consuming meals.”

**Figure 1.** Distribution of responses to the items of the scale that are considered

**Note:** p = p-value referring to Pearson’s chi-squared test.

**Figure 2.** Distribution of responses to the items of the scale that are considered

**Note:** p = p-value referring to Pearson’s chi-squared test.

Figure 2 shows the distribution of responses to the items referring to eating practices opposed to Guide recommendations (“choice of food” and “ways of eating” dimensions) by stage of life (adults and older adults), in which it is recommended that interviewees disagree with the statements (green). Significant differences occurred between the stages of life (p ≤ 0.05), showing that older adults have a higher proportion of practices in line with the Guide in all items, except for “I usually eat my meals at the work or study table.”

Considering the outcome of the prevalence of adherence to dietary practices in line with the recommendations of the Guide, Table 2 shows the results of the bivariate analysis with demographic and socioeconomic factors. Considering high adherence as the category of analysis, women tended to adhere more to Guide recommendations (p = 0.076), a result that was negatively associated with those who lacked daily access to water (p = 0.0065). Older adults showed a predominantly high adherence (p = 0.001), and the higher the level of education and *per capita* income, the higher the adherence (according to the chi-squared linear trend 4.36 (p = 0.0037) and 4.72 (p = 0.029), respectively).

**Table 3.** Multiple analysis of the prevalence of high adherence to Guide recommendations with demographic and socioeconomic characteristics of adults and older adults in the Brazuca Natal study – 2019–2020 (n = 411).

Variables/categories	Gross OR ^a^	Adjusted OR ^a^
	(95%CI)	(95%CI)
Sex		
Female	1.44 (0.96–2.15)	1.70 (1.10–2.64)
Male	1.00	1.00
Stage of life		
Older adult	2.39 (1.47–3.88)	2.27 (1.36–3.80)
Adult	1.00	1.00
Income *per capita* ^b^		
< ¼ minimum wage	0.43 (0.25–0.73)	0.25 (0.12–0.53)
≥ ¼ and < ½ minimum wage	0.94 (0.67–1.33)	0.45 (0.18–1.14)
≥ ½ and < 1 minimum wage	1.65 (1.12–2.43)	0.51 (0.27–0.97)
≥ 1 minimum wage	1.00	1.00

OR: odds ratio; 95%CI: 95% confidence interval.

^a^Values expressed in complex sampling analysis, weighted by sex, life stage, and socioeconomic status considering the census tract.

^b^Chi-squared test of linear trend = 4.72 (p = 0.029).

Table 3 shows the results of the multiple analysis with the final significant model, which confirmed that high adherence occurred more often in women, older adults, and those with *per capita* income above one minimum wage.

## DISCUSSION

This study analyzed the adherence of a sample population of adults and older adults in a Northeastern Brazilian capital to the dietary recommendations of the Guide^2^, configuring one of the first studies with such purpose in this region. We chose to evaluate high adherence to the dietary practices the Guide recommends as an outcome to understand and encourage the practices that characterize it and to know who are the people who may show greater vulnerability (low adherence). High adherence was positively associated with the female sex and older adults, whereas those with a *per capita* income of up to one fourth of the minimum wage had a lower probability of high adherence. Regarding the overall score, less than half of the population had high adherence.

The distribution of responses by life stage to the items of the scale bases some reflections. The finding in which the adults disagreed more than older adults regarding the sentence “I usually eat fruit for breakfast” shows a convergence with the 2017–2018 POF finding: the consumption of fruits and vegetables at this stage of life decreased in relation to previous years and remains far below the recommended^8,9^.

Individuals (both adults and older adults) greatly disagreed with the sentences “In my house it is common to use whole wheat flour” and “I usually consume peas, lentils, or chickpeas instead of beans.” This result may stem from a regional particularity: the habit of consuming these foods is uncommon in the Brazilian Northeast. However, a variety of natural and minimally processed foods in this region may reflect an adequate intake of fiber—cassava flour, couscous, and regional roots, for example—and legumes given the several types of beans. The Brazilian Northeast has a characteristic cuisine that offers good sources of nutrients, especially legumes (mesquite, beans, cowpeas, green beans, pigeon peas), tubers, roots, cereals (arrowroot, sesame, yams, sedge, cassava, sorghum), and flours (tapioca flour)^20^. The Guide adherence scale considers the diversity of legumes, offering examples such as chickpeas, peas, and lentils, which may fail to reflect local habits. Thus, the answers to this question may fail to reflect the reality of the Brazilian Northeast. Adjusting the guidelines to apply this scale considering the food particularities of the Brazilian regions could enhance this important tool.

A positive factor refers to the high percentage of agreement, especially by older adults, to the items “I try to eat meals slowly” and “At home, we share tasks that involve the preparation and consumption of meals,” signaling the importance given to the ways of eating and sharing meals at this stage of life. When adults agree more than older adults with some practices related to inappropriate behaviors and ways of eating, such as “I usually eat my meals sitting on the living room sofa or in bed,” “I take advantage of meal times to deal with other things and end up skipping meals,” and “I usually snack between meals” show some possible obstacles to the adoption of a healthier diet in adults, such as lack of time to organize and prepare food, lack of cooking skills, or the overload of tasks related to food on a single person in the family. Inappropriate eating such as eating while performing other activities, lack of a chosen space to have meals, and steering away from culinary tradition can easily favor the consumption of UPF, which are designed to be accessible, convenient, and hyper-palatable^2,18,21^.

A considerable percentage of these adults also agree more than older adults with questions related to UPF consumption, such as “I usually drink soda,” “I usually drink industrialized juices, such as boxed, powdered, bottled, or canned,” and “I usually eat candies, chocolates, and other sweets.” According to data from the 2017–2018 POF^8^, UPF contribute to 19.5% of the calories Brazilian adults consume, especially salty biscuits and packaged snacks, industrialized breads, sweet and cold biscuits, and processed meats. The high percentage of older adults also agreed with the statements “When I drink coffee or tea, I usually add sugar” and “I usually eat candies, chocolates, and other sweets.” UPF contribute to about 15% of the calories these people consume, especially crackers and industrialized breads, followed by sweets^8^.

The high adherence in older adults and the lower adherence in those with a *per capita* income of up to one fourth of the minimum wage resemble Gabe and Jaime^2^, which also found a direct association between compliance with Guide recommendations and increasing age and a trend toward a decrease in adherence from the highest to the lowest economic classes. The linear increase in age and better eating practices may reflect socioeconomic factors (fixed and permanent income from retirements, pensions, and social benefits), lifestyle, greater availability to prepare meals, or even the presence of chronic diseases and comorbidities that require the adoption of healthier eating habits. The multidimensional health of older adults shows that improving one dimension (diet, for example) fails to necessarily do so in another^22^.

The greater probability in this study of high adherence to Guide recommendations in women suggests that the lifestyle and attitudes related to women’s food choices can influence eating practices. A previous study has found that protective behavior toward healthy eating patterns occurred more often in White and older women^23^. Culinary skills among women can also influence this outcome. Another study has found a higher consumption of fruit in women, showing an inversely proportional relationship with UPF intake^24^.

The finding that relates people with lower *per capita* income to a lower prevalence of high adherence to the Guide evinces the importance of policies to improve the living conditions of these people. In Brazil, although the consumption of UPF is lower in the lower quintiles of family income, these foods permeate all social strata^9^. The difficulty of acquiring basic healthy items such as fruits, vegetables, whole foods, and oilseeds can directly interfere with the adoption of a healthy diet^25^. Food consumption includes the quality of the food, which suffers the influence of cultural, historical, and psychological characteristics that are linked to low income^9^.

The dismissal of older adults in the development and validation of the scale of adherence to Guide recommendations configures an inherent limitation of this instrument. Another limitation refers to the homogeneity of this population regarding *per capita* income since most participants in this study have low income. Moreover, the design effect values for some estimates may indicate a lower precision of the estimates and differ across outcome. This may have occurred due to the suspension of data collection due to COVID-19. However, these facts fail to invalidate our findings since we showed the peculiarities of the instrument for older adults and regarding the cultural habits of a Northeastern Brazilian capital. We also carefully studied loss analysis. Another point to be considered refers to our choice of using the authors’ cutoff points for the scale of adherence to the Guide, thus considering the percentiles of another sample. However, we stress that this fails to invalidate our findings since the scale has been validated for Brazilian adults and older adults^26^.

We highlight another potential of this study: the violation of the human right to adequate and healthy food and other rights for most of the population living in low income in the municipality before the pandemic. Moreover, this is one of the first studies in Northeastern Brazil to use the multidimensional scale to assess eating practices according to the Guide.

It showed better adherence to the Guide in women and older adults, who usually concern themselves and care more for their health in Brazil. This study also showed that lower income seems to determine worse adherence to Guide recommendations. This research corroborates the potential of this tool to guide public policies to promote healthy eating and may usefully direct local and national actions to disseminate the Guide.
